# Rearrangements of the Actin Cytoskeleton and E-Cadherin–Based Adherens Junctions Caused by Neoplasic Transformation Change Cell–Cell Interactions

**DOI:** 10.1371/journal.pone.0008027

**Published:** 2009-11-30

**Authors:** Dmitry V. Ayollo, Irina Y. Zhitnyak, Jury M. Vasiliev, Natalya A. Gloushankova

**Affiliations:** Institute of Carcinogenesis, N.N. Blokhin Cancer Research Center of the Russian Academy of Medical Sciences, Moscow, Russia; LMU University of Munich, Germany

## Abstract

E-cadherin–mediated cell–cell adhesion, which is essential for the maintenance of the architecture and integrity of epithelial tissues, is often lost during carcinoma progression. To better understand the nature of alterations of cell–cell interactions at the early stages of neoplastic evolution of epithelial cells, we examined the line of nontransformed IAR-2 epithelial cells and their descendants, lines of IAR-6-1 epithelial cells transformed with dimethylnitrosamine and IAR1170 cells transformed with N-RasG12D. IAR-6-1 and IAR1170 cells retained E-cadherin, displayed discoid or polygonal morphology, and formed monolayers similar to IAR-2 monolayer. Fluorescence staining, however, showed that in IAR1170 and IAR-6-1 cells the marginal actin bundle, which is typical of nontransformed IAR-2 cells, disappeared, and the continuous adhesion belt (tangential adherens junctions (AJs)) was replaced by radially oriented E-cadherin–based AJs. Time-lapse imaging of IAR-6-1 cells stably transfected with GFP-E-cadherin revealed that AJs in transformed cells are very dynamic and unstable. The regulation of AJ assembly by Rho family small GTPases was different in nontransformed and in transformed IAR epithelial cells. As our experiments with the ROCK inhibitor Y-27632 and the myosin II inhibitor blebbistatin have shown, the formation and maintenance of radial AJs critically depend on myosin II-mediated contractility. Using the RNAi technique for the depletion of mDia1 and loading cells with N17Rac, we established that mDia1 and Rac are involved in the assembly of tangential AJs in nontransformed epithelial cells but not in radial AJs in transformed cells. Neoplastic transformation changed cell–cell interactions, preventing contact paralysis after the establishment of cell–cell contact and promoting dynamic cell–cell adhesion and motile behavior of cells. It is suggested that the disappearance of the marginal actin bundle and rearrangements of AJs may change the adhesive function of E-cadherin and play an active role in migratory activity of carcinoma cells.

## Introduction

Analyses of cell-cell interactions and of the establishment of cell-cell adhesion are important directions in the studies of normal morphogenesis and its pathological alterations during tumor progression. Long ago, Abercrombie coined the term ‘contact inhibition of locomotion’ [1] for a phenomenon that has recently been described for neural crest cell migration *in vivo* [2]. The nature of this phenomenon and of changes of cell-cell interactions of neoplastic cells has still not been elucidated. Classical cadherins are transmembrane proteins that mediate cell-cell adhesion through Ca^+2^-dependent homophilic interactions of their ectodomains. The intracellular domain of the cadherin molecule interacts with proteins of cytoplasmic plaque (catenins, vinculun) that link it to actin filaments to maintain the stability of adherens junctions (AJs). The stable E-cadherin-based AJs play a pivotal role in the integrity of the epithelium and the maintenance of tissue homeostasis [3]. Cell-cell adhesion is often modified during cancer progression [4]. Epithelial-mesenchymal transition (EMT), which includes the downregulation of E-cadherin expression, the disruption of cell-cell contacts, and the acquisition of an elongated fibroblast-like phenotype by epithelial cells, has been observed in many carcinoma cell lines. It has been thought for a long time that a decrease in the E-cadherin level on the cell surface in the course of EMT is a key step of the progression from adenoma to carcinoma [5]. Many tumors, however, continue to express E-cadherin [6], and thus there should be other mechanisms to regulate the E-cadherin adhesive function in carcinomas. The early stages of the morphological transformation of epithelial cells, including rearrangements of the actin cytoskeleton and cell-cell interactions, have not yet been characterized.

It has been shown that Rho family small GTPases, which regulate cytoskeletal dynamics [7], are involved in cadherin-dependent adhesion. In epithelial cells, inhibition of endogenous Rho or Rac decreased accumulation of E-cadherin at cell-cell contacts [8], [9]. Rac1, which regulates Arp2/3-mediated actin polymerization, is associated with the newly formed cell-cell contacts [10]. Early AJs incorporate exogenous G-actin and depend on actin polymerization [11], [12]. The Arp2/3 regulators N-WASP, WAVE2, and cortactin are involved in the assembly of AJs [12]–[15]. Rho activity is required for the formation of actin stress fibers and focal contacts [7]. Rho-kinase (ROCK) and the formin protein mDia1 are the main effectors of Rho [16]–[18]. Active Rho recruites ROCK, which stimulates acto-myosin contractility by phosphorylation of the myosin II light chain and other targets [19]. Data on the role of myosin II-mediated contractility in the establishment of cadherin-mediated adhesion are controversial. Shewan et al. [20] suggested that myosin II is essential for the accumulation of E-cadherin at cell-cell junctions. In other studies, the ROCK inhibitor Y-27632 and the myosin II ATPase inhibitor blebbistatin had no effect on the assembly of AJs in epithelial cells [12], [21]. Formin-mediated actin polymerization could also be important for the establishment of AJs. Formin1 is essential for cadherin-mediated adhesion in keratinocytes [22]. It has also been shown that mDia1 participates in the formation and maintenance of AJs in MCF7 epithelial cells [23].

In this work, we studied the lines of transformed epithelial cells that retained expression of E-cadherin, displayed slightly changed morphology, and formed monolayers in dense culture. In transformed epithelial cells, we found, however, a disappearance of the marginal actin bundle and dramatic rearrangement of E-cadherin-based AJs into radial AJs that are differently regulated by Rho family small GTPases than are tangential AJs in nontransformed epithelial cells. We also described the remodeling of radial AJs and the alterations in protrusive activity that led to reduced cell-cell adhesion and to acquisition of motile behavior and migratory capacity by transformed epithelial cells.

## Results

### Actin cytoskeleton and AJs in nontransformed IAR-2 epithelial cells and transformed IAR descendants

**Figure 1:**
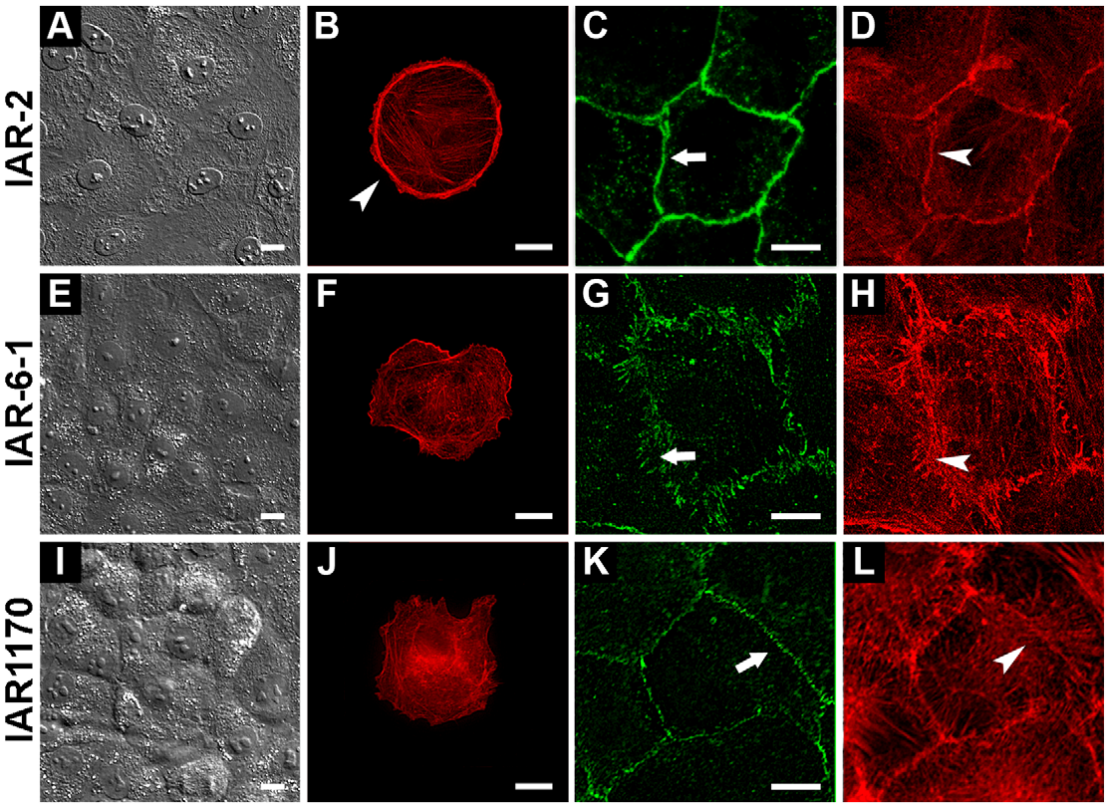
Nontransformed and transformed IAR epithelial cell lines. (A) In dense culture, nontransformed IAR-2 epithelial cells form a monolayer. (B) A characteristic feature of the actin cytoskeleton of an IAR-2 epithelial cell is the marginal actin bundle (arrowhead). (C, D) E-cadherin-based AJs (tangential AJs) (arrow) in an IAR-2 monolayer organized into adhesion belts along the cell-cell boundaries and colocalized with perijunctional actin bundles (arrowhead). (E–H) An IAR-6-1 line transformed with dimethylnitrosamine. (E) In dense culture, transformed IAR-6-1 epithelial cells form a monolayer. (F) IAR-6-1 cells have a slightly changed morphology. They are polygonal. Note the disappearance of the marginal actin bundle at the cell periphery. (G, H) Double staining for E-cadherin and F-actin shows that AJs in IAR-6-1 cells appear as radial strands (arrow) that are colocalized with straight actin bundles (arrowhead). (I–L) Ras-transformed IAR1170 epithelial cells (clone H5). (I) In dense culture, IAR1170 cells form a monolayer. (J) The shapes of IAR1170 cells in sparse cultures are polygonal. The actin cytoskeleton is present in the form of randomly oriented actin bundles; the marginal actin bundle disappears. (K–L) Punctate clasters of E-cadherin at the cell-cell boundaries in an IAR1170 monolayer (arrow). Circumferential actin bundles are disorganized. Straight actin bundles oriented perpendicularly to the cell-cell boundaries (arrowhead) are seen. Bar, 10 µm.

In this study, we compared the morphology, the actin cytoskeleton, and AJs of nontransformed IAR-2 epithelial cells derived from rat liver with those of the transformed IAR descendants IAR-6-1 and IAR1170. In dense culture, nontransformed IAR-2 epithelial cells formed a monolayer (Figure 1A). Individual IAR-2 cells were discoid-shaped. They had a marginal actin bundle, which is typical of epithelial cells and internal straight actin bundles (Figure 1B). Immunofluorescence microscopy of IAR-2 cells stained with antibodies against E-cadherin showed that AJs were aligned as a continuous belt along the cell-cell boundaries and colocalized with the circumferential actin belt (Figure 1C and 1D). We designated AJs of nontransformed epithelial cells ‘tangential AJs’.

Most descriptions of transformed epithelial cells *in vitro* focus on cell lines that underwent EMT. We, however, decided to study transformed epithelial cell lines that had a slightly changed morphology. We chose an IAR epithelial line transformed *in vitro* with dimethylnitrosamine (IAR-6-1). As previously reported [35], the IAR-6-1 line was tumorigenic in syngenic rats. In comparison with IAR-2 cells, the morphology of IAR-6-1 cells was minimally changed. Single IAR-6-1 cells were polygonal or discoid. In confluent culture, IAR-6-1 cells formed a monolayer similar to the IAR-2 monolayer (Figure 1E and 1F). Western blot analysis showed that transformed IAR-6-1 epithelial cells retained expression of epithelial E-cadherin and did not express mesenchymal N-cadherin (Figure S1). Differences between nontransformed IAR-2 cells and transformed IAR-6-1 cells became evident when they were fluorescently stained for the actin cytoskeleton and E-cadherin. In contrast with nontransformed IAR-2 cells, IAR-6-1 cells had only randomly oriented actin bundles in the cytoplasm; the marginal actin bundles had disappeared (Figure 1F). We also revealed the dramatic rearrangement of E-cadherin-based AJs in IAR-6-1 cells that were organized into strands arranged perpendicularly or at different angles to the cell edge (Figure 1G). We designated AJs of transformed epithelial cells ‘radial AJs’. Double fluorescence staining showed that AJs in transformed IAR-6-1 cells were colocalized with short straight actin bundles (Figure 1G and 1H). In dense cultures, some AJs were oriented along the cell-cell borders.

We also established 14 clones of IAR-2 cells transformed with Ras and compared their morphology. Cells of nine Ras-transformed clones (IAR1162 clones) underwent EMT: they lost the epithelial phenotype and acquired fibroblastic-like morphology. As Western blotting showed, IAR1162 cells did not expressed epithelial E-cadherin and did express mesenchymal N-cadherin (data not shown). We investigated, however, the other five clones of Ras-transformed IAR-2 cells (IAR1170-D6, -D11, -F9, -H5, and -D12), which in comparison with nontransformed IAR-2 cells had slightly changed morphology. Ras-transformed IAR1170 cell lines were tumorigenic in nude mice (Figure S2). All five clones had similar morphology. The shapes of IAR1170 cells were near-discoid or polygonal. In confluent culture, IAR-1170 cells formed monolayers. As an example, we give images of IAR1170-H5 clone (Figure 1I and 1J). Western blot analysis showed that cells of IAR1170 clones retained E-cadherin and also expressed N-cadherin (Figure S1). A characteristic feature of the actin cytoskeleton in IAR1170 cells was the disappearance of the marginal actin bundle (Figure1J). In IAR1170 cells, we also revealed the reorganization of E-cadherin-based AJs. The continuous adhesion belt that was typical of IAR-2 cells was destroyed. AJs stained for E-cadherin were seen as punctate clusters at the cell-cell boundaries. The circumferential actin belts were disorganized (Figure 1K and 1L).

Thus, we described the early stages of the morphological transformation of epithelial cells, when cells retained expression of E-cadherin, had discoid or polygonal morphology, and formed monolayers. Neoplastic transformation, however, led to the disappearance of the marginal actin bundle and to the dramatic rearrangement of E-cadherin-based AJs.

### Alterations of cell–cell interactions and promotion of motile behavior of epithelial cells caused by neoplastic transformation

**Figure 2:**
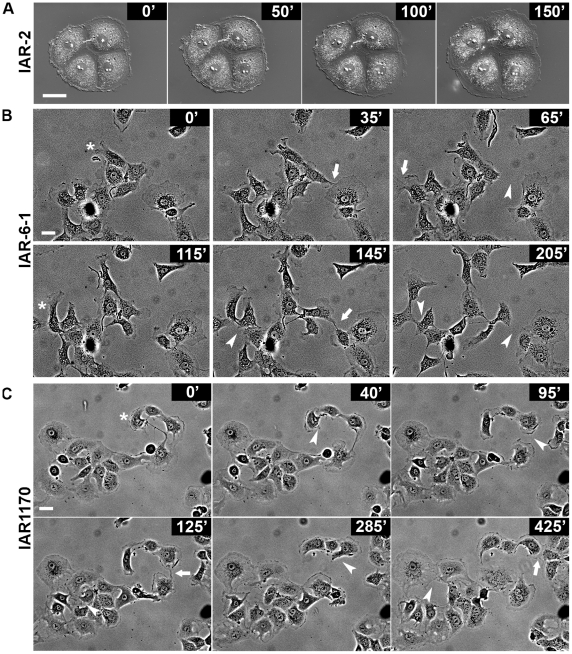
The alterations of motile behavior of transformed IAR-6-1 and IAR1170 cells in comparison with those of nontransformed IAR-2 cells. (A–C) Selected frames from the time-lapse sequences of sparse cultures showing cell behavior and cell-cell interactions. (A) Nontransformed IAR-2 cells form an island. Cell-cell contacts are stable during the entire period of observation (see also Video S1). (B) Transformed IAR-6-1 cells; (C) transformed IAR1170 cells (clone H5). In cultures of transformed IAR-6-1 and IAR1170 cells, significant alterations in cell-cell interactions are seen. Cells extend lamellipodia at different sites of the free edges and establish contacts with different neighboring cells (arrows). These contacts are unstable and often break (arrowheads). Cells could move in different directions (asterisks) (see also Video S2 and Video S3). Bar, 20 µm.

As neoplastic transformation leads to disappearance of the marginal actin bundle and to rearrangements of AJs in epithelial cells, we decided to test whether these alterations contribute to cell-cell interactions of transformed IAR-6-1 and IAR1170 cells. To compare cell-cell interactions in cultures of nontransformed and transformed cells, sparse cultures of IAR-2, IAR-6-1, and IAR1170 cells were observed with time-lapse videomicroscopy. Nontransformed IAR-2 cells in sparse cultures formed islands. Time-lapse imaging showed that cell-cell contacts in these islands were stable (Figure 2A and Video S1) and were disrupted only during mitosis. Contacts between neighboring islands or single IAR-2 cells led to the establishment of strong cell-cell adhesion and development of a continuous monolayer for 2–3 days.

In cultures of transformed IAR-6-1 and IAR1170 cells, we revealed significant alterations in cell-cell interactions. In contrast with nontransformed IAR-2 cells, cell-cell contacts in cultures of transformed IAR-6-1 and IAR1170 cells were unstable and often broke (Figure 2B and 2C, Video S2, and Video S3). Transformed cells in contact extended lamellipodia at free edges. During 6–7 h of observation, the same cell could establish and break contacts with different neighboring cells and could move in different directions. This behavior of transformed epithelial cells may promote their migratory activity.

**Figure 3:**
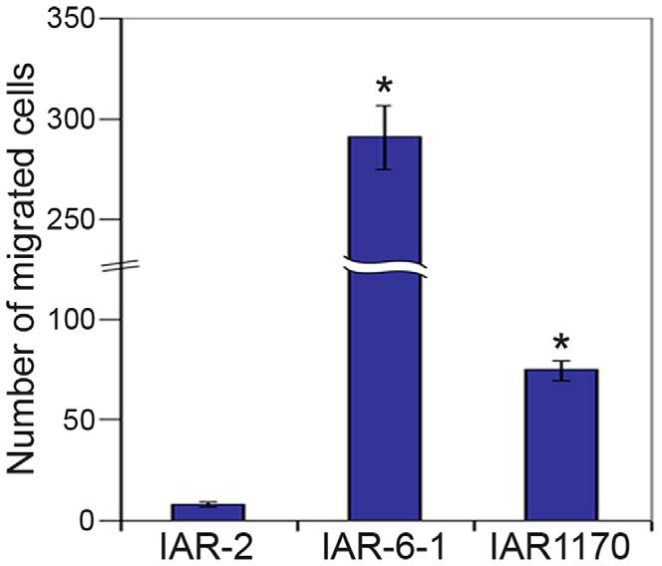
Transformed IAR-6-1 and IAR1170 cells display enhanced motility in a migration assay. IAR-2, IAR-6-1, and IAR-1170 (clone H5) cells were plated on membranes with 8-µm pores in Bio-Coat migration chambers. After 20 h of incubation, the cells on the lower surface of membranes were fixed and stained. The number of migrated cells was determined from an average of the number of stained cells on membranes in 15 randomly selected fields. The data are presented as means±SEM of triplicate assays for each cell line in three independent experiments. Asterisks indicate the values that differ significantly from control (*p*<0.001, *t*-test).

To explore the effects of changed motile behavior of transformed IAR-6-1 and IAR1170 cells on their migration, the ability of cells to migrate through polycarbonate membrane inserts with 8-µm pores in Bio-Coat migration chambers was examined. Migration assay showed that the number of migrating IAR-6-1 and IAR1170 cells was higher than that among nontransformed IAR-2 cells (Figure 3). Thus, disappearance of the marginal actin bundle and rearrangement of E-cadherin-based AJs is accompanied by reduction in cell-cell adhesion and promotion of migratory activity of transformed cells.

### Analysis of the dynamics of cell–cell contact formation in a narrow wound

**Figure 4:**
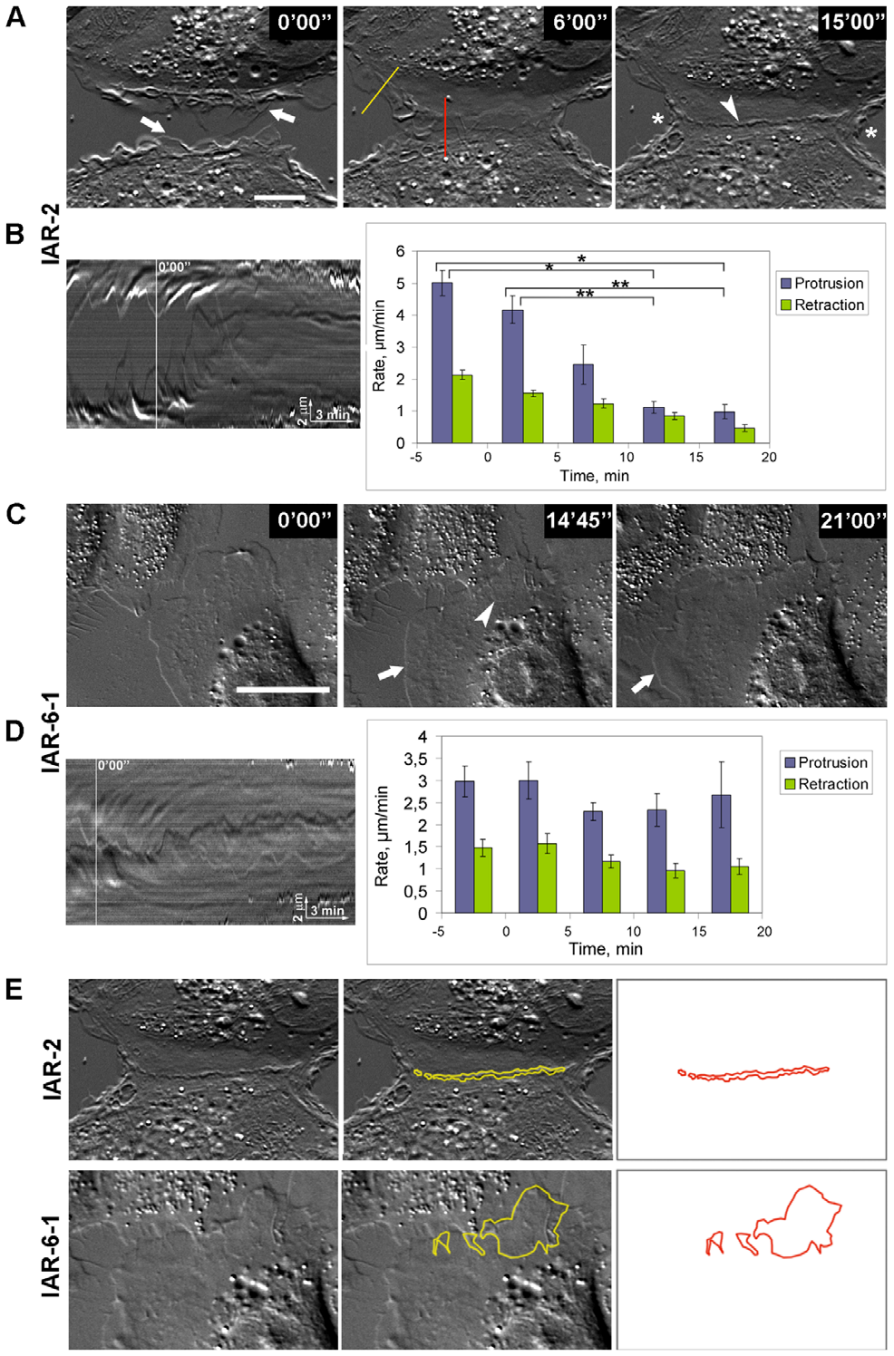
Dynamics of cell-cell interactions in a narrow wound. Time-lapse images of colliding cells. (A, B) Nontransformed IAR-2 cells extend lamellipodia (arrows) and establish a cell-cell contact that expands laterally. By 15–20 min, protrusion of lamellipodia at the site of the contact is inhibited (contact paralysis) (arrowhead) (see also Video S4). Protrusive activity at the free edges of contacting cells also decreases (asterisks). Kymographs were generated along straight lines in different regions. (B) Left: kymograph generated at the site of the cell-cell contact (red line in A) shows contact paralysis of protrusive activity by 15–20 min after the establishment of the contact. White line on kymograph indicates the time of the establishment of the contact. Right: quantification of the rates of lamellipodial protrusion and retraction at the sites of cell-cell contacts. Data are presented as means±SEM. Brackets indicate statistically significant differences between the mean rates of lamellipodial protrusion (*, *p*<0.001; **, *p*<0.005, *n* = 25 cells, *t*-test). (C, D) Transformed IAR-6-1 cells. During formation of the cell-cell contact, protrusive activity persists both at the site of the contact (arrowhead) and at the free edges, where new lamellipodia are formed (arrows) (see also Video S5). (D) Left: kymograph generated at the site of the contact (left) shows the disappearance of contact paralysis. White line on kymograph indicates the time of the establishment of the contact. Right: the rates of lamellipodial protrusion and retraction at the sites of cell-cell contacts did not change within the time of observation. Data are presented as means±SEM. (E). Measurement of overlapping areas of contacting cells (15 min after formation of cell-cell contact). In cultures of transformed IAR-6-1 cells, there is prominent overlapping of lamellae of contacting cells. Bar, 10 µm.

For detailed analysis of cell behavior during formation of contact with neighboring cells, nontransformed IAR-2 cells and transformed descendants were imaged in a narrow wound (∼100 µm) in a monolayer. As IAR1170 expressed both E- and N-cadherin and there are findings that N-cadherin promotes motility when expressed by epithelial cells [24], for detailed studies we focused on IAR-6-1 cells, which expressed E-cadherin and did not express mesenchymal N-cadherin. We made narrow wounds by scraping cell monolayers with a sterile needle. After 3–4 h of incubation, cells from adjacent monolayers began to form new cell-cell contacts in the area of colliding lamellipodia. In the case of nontransformed IAR-2 cells, there was lateral expansion of initial stable contact between opposing cells (Figure 4A and Video S4). During the first 5–10 min after the establishment of a stable contact, cells formed lamellipodia both in the zone of contact and at the free edges. Within the next 15–20 min, dramatic cessation of protrusive activity along the entire contact (contact paralysis) developed. It began simultaneously along the entire length of the contact. The cells formed small lamellipodia only at the edges of the contact. Contact paralysis did not extend to the free edges of contacting cells. We observed, however, a decrease of protrusive activity at the free edges of contacting cells. We analyzed protrusive activity using kymographs (Figure 4B). Quantitative analysis of kymographs showed that both phases of the protrusion-retraction cycle, especially protrusions, were affected during contact paralysis. The rate of lamellipodial protrusion at the site of the contact was strongly diminished by 15–20 min after the establishment of the contact. The rate of lamellipodial retraction at the site of the contact was also reduced. Analysis of kymographs also showed statistically significant decrease of the mean rate of lamellipodial protrusion at the free edges of contacting cells. (Figure S3).

Neoplastic transformation modified the dynamics of cell-cell interactions in a narrow wound. After the establishment of the contact between two cells, transformed IAR-6-1 cells continued to form lamellipodia both at the site of the contact and at the free edges (Figure 4C and Video S5). The rates of lamellipodial protrusion and retraction did not change (Figure 4D and Figure S3). We did not see contact paralysis of protrusive activity at the site of the contact of IAR-6-1 cells (Figure 4D), and further analysis revealed significant overlapping in this zone (Figure 4E). While the mean overlapping area of contacting IAR-2 cells was 18.0±2.93 µm^2^ (mean±SEM, *n* = 12), the mean overlapping area of contacting IAR-6-1 cells was 76.5±17.37 µm^2^ (*n* = 14) at 15 min after cell-cell contact establishment.

The studies performed demonstrated the differences in cell behavior during the establishment of cell-cell contact by nontransformed and transformed cells. Cell-cell interactions of nontransformed epithelial cells result in the establishment of stable cell-cell contact followed by contact paralysis at the site of the contact and a decrease of protrusive activity at the free edges of the contacting cells. Neoplastic transformation changes cell-cell interactions, preventing contact paralysis after the establishment of cell-cell contact, leading to overlapping of lamellae and to the extension of lamellipodia at the free edges.

### ynamics of E-cadherin–based AJs in nontransformed and transformed cells

**Figure 5:**
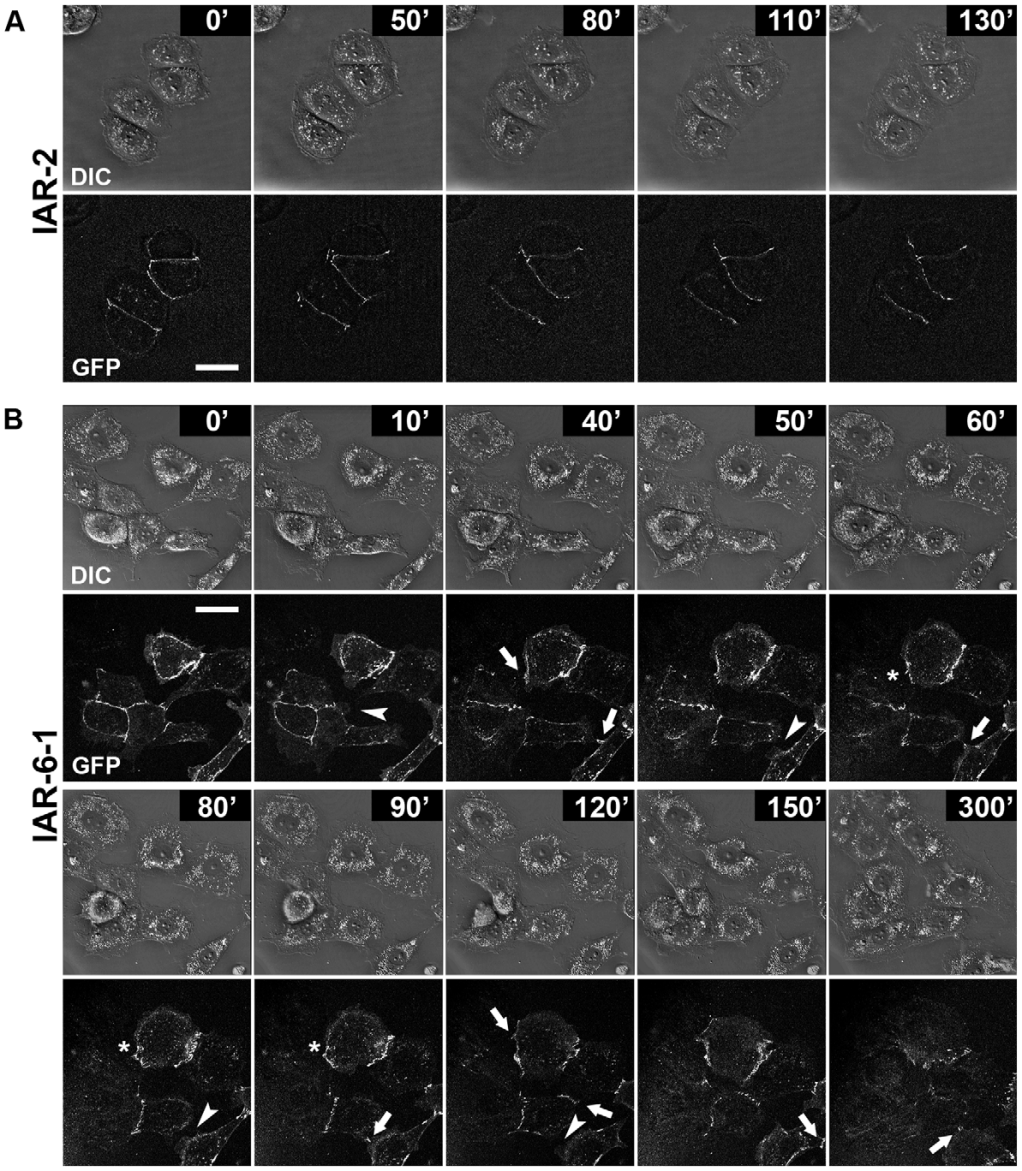
E-cadherin dynamics at the sites of cell–cell contacts. Nontransformed IAR-2 cells and transformed IAR-6-1 cells were stably transfected with GFP-E-cadherin. (A) In IAR-2 cells, GFP-E-cadherin accumulates in the zones of cell-cell interactions, forming stable tangential AJs (see also Video S6). (B) IAR-6-1 cells form lamellipodia along their entire edges. When a protrusion contacts another cell, E-cadherin begins to aggregate in punctate AJs in the zone of contact (arrows). AJs can grow, rearrange, and relocate (asterisks). The disruption of AJs results in detachment of one cell from another (arrowheads) (see also Video S7). Bar, 10 µm.

To correlate cell behavior with the dynamics of junctional E-cadherin, we established nontransformed IAR-2 cells and transformed IAR-6-1 cells stably expressing GFP-E-cadherin. Using time-lapse differential interference contrast (DIC) and fluorescence microscopy, we investigated living cells for 3–7 h. In nontransformed IAR-2 cells, GFP-E-cadherin accumulated in stable continuous tangential AJs at the cell-cell boundaries (Figure 5A and Video S6). When IAR-2 cells formed new cell-cell contacts, GFP-E-cadherin assembled in tangential lines along the cell-cell boundaries. In contrast, in transformed IAR-6-1 cells, AJs were dynamic (Figure 5B and Video S7). Transformed cells extended protrusions over the entire periphery. When a protrusion contacted another cell, GFP-E-cadherin began to accumulate in dot-like clusters at the site of the cell-cell contact, which were able to grow. In IAR-6-1 cells, we found the remodeling of AJs. AJs in IAR-6-1 cells could rearrange and relocate in the zone of contact. Unlike stable AJs in IAR-2 cells, AJs in IAR-6-1 cells were often disrupted. As a cell detached from one cell, it could form a contact with another cell, in which GFP-E-cadherin accumulated. Thus, transformed IAR-6-1 cells could move and establish dynamic E-cadherin-based cell-cell contacts with neighboring cells.

Using the distance map method [25], we analyzed the dynamics of AJs in IAR-2 cells and IAR-6-1 cells stably expressing GFP-E-cadherin (Figure S4). Time-lapse sequences of sparse cultures were used to calculate the mean rates of AJ movement. We found differences in the dynamics of AJs in IAR-2 cells and IAR-6-1 cells. While the mean rate of movement of tangential AJs in IAR-2 cells was 59±3 nm/min (mean±SEM, *n* = 28), the mean rate of movement of radial AJs in IAR-6-1 cells was 189±21 nm/min (*n* = 30). Using ImageJ software, we also measured the distances traveled by individual AJs, and calculated the mean rates of AJ movement. This analysis showed similar results: the mean rate of movement of AJs in IAR-2 cells was 97±17 nm/min (mean±SEM, *n* = 25), the mean rate of movement of radial AJs in IAR-6-1 cells was 176±22 nm/min (mean±SEM, *n* = 25).

**Figure 6:**
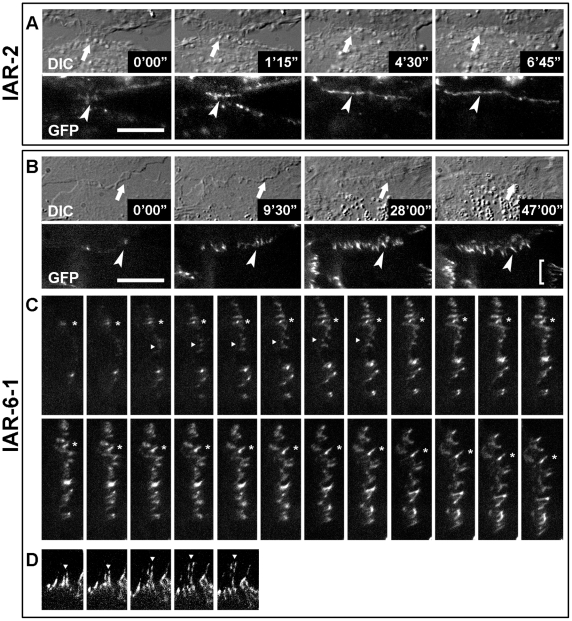
E-cadherin dynamics during cell–cell collision in a narrow wound. Nontransformed IAR-2 cells and transformed IAR-6-1 cells were stably transfected with GFP-E-cadherin. (A) IAR-2 cells. DIC image shows lateral expansion of the cell-cell contact (arrows). During cell-cell contact formation, GFP-E-cadherin accumulates in a tangential line (arrowheads) at the boundary between two cells (see also Video S8). (B, C, D; see also Video S9) IAR-6-1 cells. (B) During cell-cell contact formation, there is overlapping of lamellae in the area of the contact (arrows). Radial AJs (arrowheads) assemble in the zone of overlapping. (C) Time-lapse images of the same cell-cell contact acquired at 1-min intervals. GFP-E-cadherin initially aggregates into dot-like clusters. A majority of GFP-E-cadherin dots grow and elongate (asterisks). Other AJs can disappear (arrowheads). (D) Because of cell movement, pre-existing AJs (bracket in B) elongate and break (arrowheads). Interval time, 2 min. Bar, 10 µm.

To compare the dynamics of AJ assembly in nontransformed and transformed epithelial cells thoroughly, we investigated cell-cell collisions in a narrow wound. Live-cell imaging of IAR-2 cultures showed that during the establishment of the cell-cell contact, a tangential line of GFP-E-cadherin formed at the cell-cell boundary and expanded laterally in two opposite directions (Figure 6A and Video S8). The characteristics of GFP-E-cadherin accumulation at the sites of cell-cell contacts in cultures of IAR-6-1 cells were different (Figure 6B, 6C, and 6D, and Video S9). At the site of the cell-cell contact, GFP-E-cadherin initially aggregated into dot-like clusters. Some of these puncta could disappear. The majority of GFP-E-cadherin clusters grew and transformed into radial strands oriented perpendicularly to the cell-cell boundary. AJs could change their position. During cell displacement, AJs between two adjacent cells in a monolayer often elongated and broke. Thus, our studies revealed two different types of E-cadherin-based AJs: stable tangential AJs in nontransformed epithelial cells and relatively dynamic radial AJs in transformed epithelial cells.

### Effect of C3 loading on the formation of different types of AJs

To elucidate the mechanisms of the formation of different types of AJs and the rearrangement of tangential epithelial AJs into radial ones during neoplastic transformation, we examined the involvement of the Rho family small GTPases, Rho and Rac, in regulating cell-cell adhesion. Earlier, we had described radial AJs in Rat-1 fibroblasts associated with short straight actin bundles. As it has been shown that the formation of radial AJs in fibroblasts depends on myosin II-mediated contractility [26], [27], we decided to add comparisons of the effects of inhibition of Rho or Rac signaling on radial AJs in Rat-1 fibroblasts to our studies concerning the effects of this inhibition on radial AJs in transformed IAR-6-1 epithelial cells.

**Figure 7:**
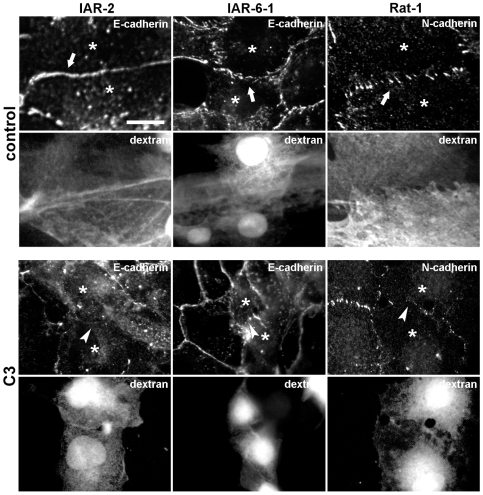
Rho activity is required for the assembly of tangential and radial AJs. Cell monolayers were wounded in the presence of C3 transferase and TRITC-labeled dextran (as a marker). Cells were fixed and stained for E-cadherin or N-cadherin. In control cultures, AJs are formed at the sites of cell-cell contacts (arrows). The IAR-2, IAR-6-1, and IAR1170 cells loaded with C3 are unable to assemble new AJs (arrowheads). Bar, 10 µm.

First, we investigated the effect of inhibition of Rho activity on AJ formation in nontransformed IAR-2 cells, transformed IAR-6-1 cells, and Rat-1 fibroblasts (Figure 7). C3, which ADP-ribosylates and which inactivates cellular Rho, was introduced into wound-edge cells during the wounding of monolayers. Wounding of a cell monolayer with a hypodermic needle in the presence of C3 and fluorescent dextran (as a marker) resulted in loading of ∼50% cells at the wound edge. Control experiments showed that fluorescent dextran alone did not affect the rate of wound healing or the accumulation of adhesive proteins in the cell-cell contacts. Loading of cultured cells with C3 led to a delay of wound healing up to 7–8 h (3–4 h in controls). Actin bundles in C3-loaded cells were disrupted (data not shown). The introduction of C3 into IAR-2 cells prevented the formation of tangential AJs. In C3-loaded IAR-2 cells, E-cadherin did not accumulate at the cell-cell boundary. Transformed IAR-6-1 cells loaded with C3 could not assemble radial AJs. E-cadherin aggregated only in dot-like clusters. Similar dot-like clusters of N-cadherin were seen at the site of the contact between C3-loaded Rat-1 fibroblasts. These cells failed to form radial AJs. Thus, Rho activity is required for the formation of tangential and radial AJs.

### Effect of the ROCK inhibitor Y-27632 and the myosin II inhibitor blebbistatin on the formation of AJs

**Figure 8:**
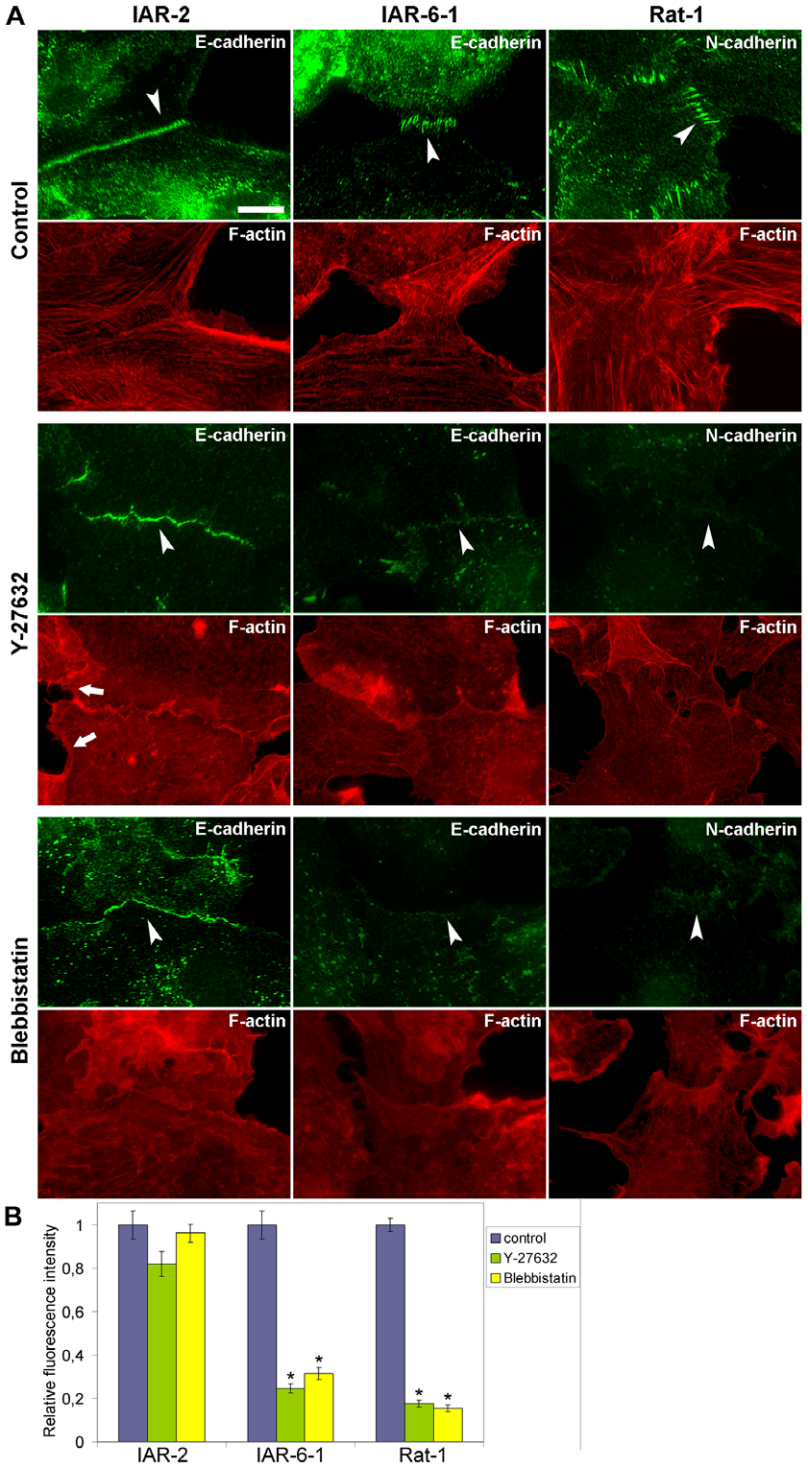
Effect of myosin II inactivation on AJ formation. Cell monolayers were wounded and transferred to the medium with the Rho-kinase inhibitor Y-27632 or with the myosin II ATPase inhibitor blebbistatin for 2 h. Cells were fixed and stained for F-actin, E-cadherin, or N-cadherin. (A) Incubation with Y-27632 (30 µM) leads to total disappearance of actin bundles. In IAR-2 cells, both marginal (arrows) and straight actin bundles disappear. IAR-2 cells can form wave-like tangential AJs (arrowhead) in the presence of 30 µM Y-27632. IAR-6-1 cells treated with 30 µM Y-27632 do not assemble radial AJs. E-cadherin-based junctions are seen as dots at the cell-cell boundary (arrowhead). Y-27632 (30 µM) blocks the formation of radial AJs in Rat-1 fibroblasts (arrowhead). Blebbistatin abolishes actin bundles in all types of cells. IAR-2 cells in the presence of 50 µM blebbistatin can accumulate E-cadherin in wave-like tangential AJs (arrowhead). Myosin II activity is necessary for the assembly of radial AJs. IAR-6-1 cells treated with 50 µM blebbistatin accumulate E-cadherin in dot-like clusters (arrowhead) and do not form radial AJs. In Rat-1 fibroblasts, blebbistatin (15 µM) inhibits the formation of radial AJs (arrowhead). (B) Accumulation of E-cadherin and N-cadherin at cell-cell contacts was quantified by measuring fluorescence intensity on immunofluorescence images. Fluorescence intensity at cell-cell contacts of drug-treated cells relative to fluorescence intensity at cell-cell contacts of control cells is shown. Vertical lines indicate SEM. Asterisks indicate the values that differ significantly from corresponding controls (*t*-test, *p*<0.001, *n* = 25–32 contacts). Bar, 10 µm.

To assess the impact of Rho effectors in the establishment of AJs of different types, we first compared the effect of the specific ROCK inhibitor Y-27632 on the formation of tangential AJs in nontransformed IAR-2 cells and of radial AJs in transformed IAR-6-1 cells and Rat-1 fibroblasts (Figure 8A). Addition of 30 µM Y-27632 to culture medium for 2–3 h resulted in disassembly of both marginal and straight actin bundles in IAR-2 cells. Nevertheless, in the presence of Y-27632, E-cadherin accumulated in tangential AJs that had a more wave-like shape than AJs in control IAR-2 cells. In the presence of Y-27632, the establishment of cell-cell contact was not accompanied by contact paralysis of colliding cells. Treated with Y-27632, IAR-2 cells continued to form lamellipodia at the sites of cell-cell contacts (Figure S5). In the case of transformed IAR-6-1 cells, 30 µM Y-27632 abolished actin bundles. Radial E-cadherin-based AJs did not assemble at the sites of cell-cell contacts. We saw staining of E-cadherin only in dot-like clusters. Y-27632 (30 µM) destroyed actin bundles and completely prevented the formation of radial AJs in Rat-1 fibroblasts. We were able to visualize only a few dots of N-cadherin at the sites of cell-cell collisions.

It is now well-recognized that Rho/ROCK signaling controls the creation of myosin II-driven forces. We examined the effect of the myosin II ATPase inhibitor blebbistatin [28] on the formation of tangential and radial AJs (Figure 8A). Our experiments showed that tangential contacts can assemble without myosin II activity. In the presence of 50 µM blebbistatin, which completely destroyed marginal and straight actin bundles in IAR-2 cells, E-cadherin accumulated in a wavy line oriented along the cell-cell boundary. Blebbistatin (50 µM) abolished actin bundles and prevented the formation of radial AJs in IAR-6-1 cells. In these cells, we revealed only dot-like and fragmented linear junctions at the sites of the cell-cell contacts. In Rat-1 cells, 50 µM blebbistatin had quick and strong effect on cell morphology, the actin cytoskeleton, and AJs and led to cell arborization (data not shown). In the case of Rat-1, we used a lower dose of blebbistatin (15 µM), which also destroyed actin bundles and stopped the assembly of radial AJs.

To quantify the effects of Y-27632 and blebbistatin on cadherin accumulation at AJs, fluorescence intensities of E-cadherin and N-cadherin at cell-cell contacts were measured as described previously [23]. Analysis of fluorescence intensity revealed that Y-27632 and blebbistatin dramatically reduced the accumulation of E-cadherin at AJs in IAR-6-1 cells and of N-cadherin at AJs in Rat-1 cells (Figure 8B). In contrast, we did not find significant differences between the average levels of E-cadherin fluorescence at cell-cell contacts in control and drug-treated IAR-2 cells.

The studies performed suggest that the establishment and maintenance of radial AJs both in transformed epithelial cells and in fibroblasts depend on myosin II-mediated contractility. In contrast, myosin II is not required for the assembly of tangential AJs in nontransformed epithelial cells.

### Effect of depletion of mDia1 on the formation of AJs

**Figure 9:**
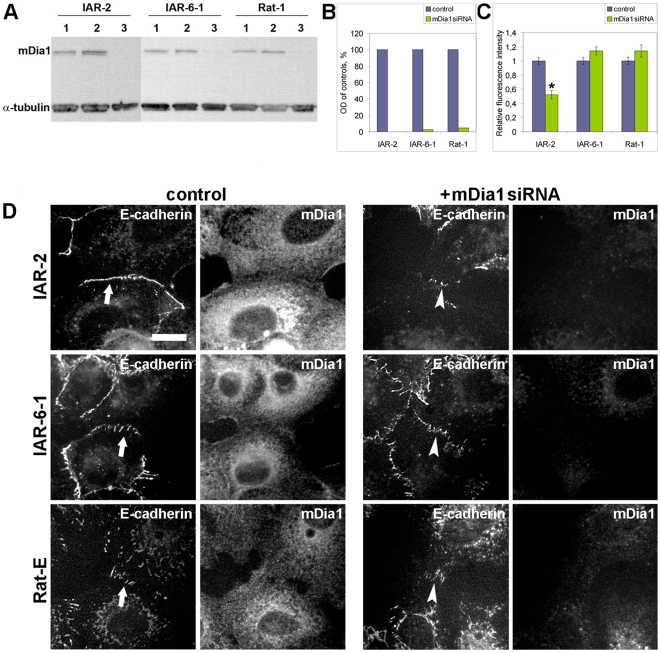
Effect of mDia1 depletion on AJ formation. Subconfluent cultures were transfected with mDia1 siRNA or control GFP siRNA and incubated for 48 h. (A, B) Immunoblot analysis of cell lysates. Total lysate protein (20 µg) was separated with SDS-PAGE and immunoblotted for mDia1 (α-tubulin was used as a loading control). Immunoblotting (A) shows the extent of mDia1 suppression by RNAi in different cell lines. Samples 1, 2, and 3 represent GFP siRNA-transfected cells, control cells, and mDia1 siRNA-transfected cells, respectively. Densitometry (B) shows the significant reduction of mDia1 after RNAi application (OD, optical density). (C) Effect of mDia1 suppression on cadherin accumulation at cell-cell contacts. Fluorescence intensity at cell-cell contacts of mDia1 siRNA-transfected cells relative to fluorescence intensity at cell-cell contacts of GFP siRNA-transfected cells on immunofluorescence images is shown. Asterisk indicates the value that differs significantly from corresponding control (*t*-test, *p*<0.001, *n* = 25 contacts). (D) Effect of mDia1 suppression on the formation of AJs in a narrow wound. Cell monolayers were wounded, fixed 4 h later, and stained for E-cadherin and mDia1 simultaneously. Arrows show the newly formed AJs in control cells. The staining for mDia1 shows a significantly lower level of mDia1 expression in the cells transfected with mDia1 siRNA than in control cells. IAR-2 cells transfected with mDia1 siRNA fail to form tangential AJs (arrowhead). mDia1 is not required for the formation of radial AJs either in transformed IAR-6-1 cells or in Rat-E fibroblasts (Rat-1 fibroblasts stably expressing E-cadherin). Radial AJs (arrowheads) are formed at the sites of contacts between mDia1 siRNA-transfected cells. Bar, 10 µm.

To investigate the involvement of another Rho effector, mDia1 formin, in the formation of AJs, we applied the RNAi technique for the depletion of mDia1 [29]. We used two types of mDia1 siRNA duplex that had similar effects. We assayed the efficiency of suppression of mDia1 using Western blot analysis of cell lysates 48 h post-transfection. Transfection with mDia1 siRNA significantly decreased the expression of mDia1 in all three cell types (Figure 9A and 9B). For double immunofluorescence staining of mDia1 and AJs, we used E-cadherin mouse monoclonal antibodies (IgG_2a_ isotype) and mDia1 antibodies (IgG_1_ isotype) revealed by secondary mouse IgG_1_ and mouse IgG_2a_ antibodies. Comparison of mDia1 immunofluorescence staining of cultures revealed a dramatic decrease of mDia1 in cells 48 h after transfection (Figure 9D). Depletion of mDia1 resulted in a prominent disturbance in the formation of tangential AJs in IAR-2 cells. In all cell pairs with diminished staining of endogenous mDia1 as a result of mDia1 suppression by RNAi, tangential AJs did not assemble. At the sites of cell-cell contacts, E-cadherin could accumulate only in dot-like clusters. These data are in agreement with the results of Carramusa et al. [23] concerning the disappearance of AJs in MCF7 epithelial cells after short-hairpin RNA knockdown of Dia1. We also compared the effects of mDia1 depletion on the formation of radial AJs in transformed IAR-6-1 epithelial cells and Rat-1 fibroblasts. Unlike tangential AJs, radial AJs assembled in mDia1 siRNA-transfected cells. Double immunofluorescence staining revealed that in IAR-6-1 cells transfected with mDia1 siRNA, E-cadherin accumulated in radial AJs. Depletion of mDia1 did not influence the assembly of radial AJs in Rat-1 fibroblasts. To demonstrate this, we established Rat-1 cells stably expressing E-cadherin that was colocalized with N-cadherin in AJs (data not shown). Using anti-E-cadherin antibodies to localize AJs in Rat-1 fibroblasts and anti-mDia1 antibodies to reveal the cells transfected with mDia1 siRNA, we found that mDia1 suppression by RNAi did not prevent the formation of radial AJs in Rat-1 fibroblasts.

To assess the influence of mDia1 depletion on cadherin accumulation at AJs, we measured the fluorescence intensities of E-cadherin at cell-cell contacts on immunofluorescence images (Figure 9C). We found that the fluorescence intensity of E-cadherin in IAR-2 cells transfected with mDia1 siRNA was reduced on average by ∼50% in comparison with that of the control. In contrast, we did not find significant differences between the fluorescence intensities of cadherins at cell-cell contacts in control and N17Rac-loaded IAR-6-1 and Rat-1 cells.

Thus, mDia1 contributes to the formation of tangential AJs in nontransformed IAR-2 epithelial cells but is not involved in the assembly and maintenance of radial AJs in transformed epithelial cells and fibroblasts.

### Effect of N17Rac loading on the formation of AJs

**Figure 10:**
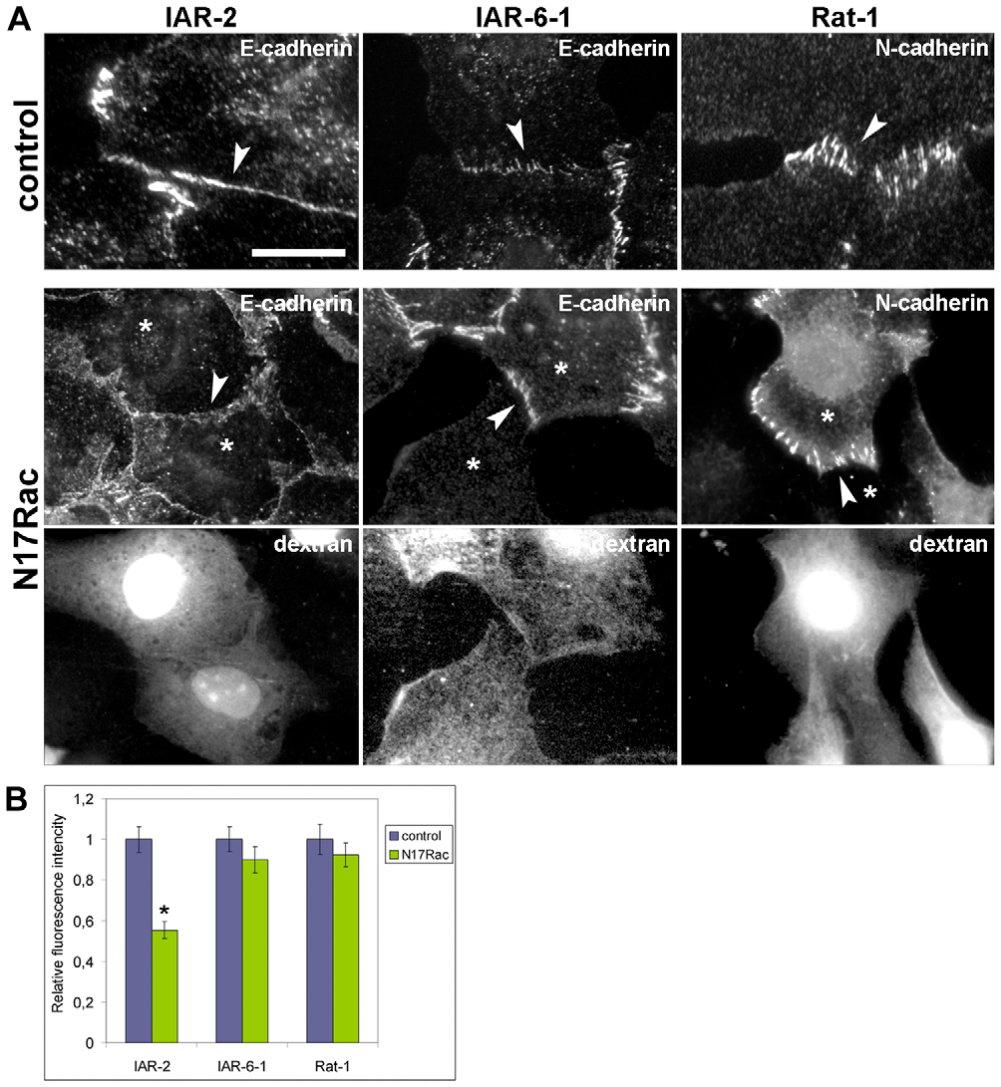
Effect of N17Rac on AJ formation in different cell types. Cell monolayers were wounded in the presence of N17Rac and TRITC-labeled dextran as a marker. (A) IAR-2 cells loaded with N17Rac fail to form continuous tangential AJs (arrowhead). Inhibition of Rac activity does not affect the formation of radial AJs. IAR-6-1 cells loaded with N17Rac assemble radial AJs (arrowhead). Rat-1 fibroblasts loaded with N17Rac also form radial AJs (arrowhead). (B) Effect of N17Rac loading on cadherin accumulation at cell-cell contacts. Fluorescence intensity of E-cadherin or N-cadherin at cell-cell contacts of N17Rac-loaded cells relative to fluorescence intensity at cell-cell contacts of control cells is shown. Asterisk indicates the value that differs significantly from corresponding control (*t*-test, *p*<0.001, *n* = 27 contacts). Bar, 10 µm.

Rac signaling has been shown to play an essential role in the establishment of epithelial AJs [8], [9]. We also examined a possible alteration of its contribution to the regulation of AJ assembly in transformed epithelial cells. A dominant-negative form of Rac, N17Rac, was loaded into cells during the wounding of monolayers to suppress the activity of cellular Rac (Figure 10A). IAR-2 cells loaded with N17Rac did not form continuous tangential AJs. In these cells, E-cadherin could accumulate only in dot-like aggregates or short linear fragments at cell-cell boundaries. In contrast, N17Rac did not affect the formation of radial E-cadherin-based AJs in transformed IAR-6-1 cells and of N-cadherin AJs in Rat-1 fibroblasts.

To assess the influence of N17Rac loading on cadherin accumulation at AJs, we also measured the fluorescence intensities of E-cadherin at cell-cell contacts in IAR-2, IAR-6-1, and N-cadherin in Rat-1 cells (Figure 10B). Analysis revealed that the fluorescence intensity of E-cadherin in N17Rac-loaded IAR-2 cells was reduced on average by ∼50% in comparison with that of the control. In contrast, we did not find significant differences between the fluorescence intensities of cadherins at cell-cell contacts in control and N17Rac-loaded IAR-6-1 and Rat-1 cells. Thus, the assembly of tangential AJs required Rac activity. In contrast, radial AJs in transformed epithelial cells and fibroblasts did not depend on functions of Rac. Collectively, the functional studies demonstrated the similarities in regulation of assembly of radial AJs in transformed epithelial cells and radial AJs in fibroblasts by Rho family small GTPases.

## Discussion

This study is the first to demonstrate that transformed epithelial cells can use the disappearance of the marginal actin bundle and the remodeling of E-cadherin-based AJs to weaken cell-cell adhesion and to acquire motile behavior and migratory activity. When we studied transformed IAR-6-1 and IAR1170 epithelial lines, we found that while cells of these lines retain E-cadherin expression, have a morphology slightly changed from the morphology of the parent nontransformed IAR-2 cells, and form monolayers in confluent culture that are similar to IAR-2 monolayer, immunofluorescence staining demonstrated dramatic rearrangements of the actin cytoskeleton and AJs. In transformed IAR-6-1 and IAR1170 cells, the adhesion belt is broken and replaced by E-cadherin-based puncta or strands resembling radial AJs of fibroblasts. AJs in transformed epithelial cells are associated with short straight actin bundles. Our data demonstrated that in contrast with stable tangential AJs in nontransformed epithelial cells, radial E-cadherin-based AJs in transformed cells are very dynamic.

To elucidate the mechanisms of formation of tangential and radial AJs, we next analyzed the involvement of small Rho GTPases and their effectors in AJ assembly. Our functional studies revealed the differences in the main driving forces of AJ formation in nontransformed and transformed epithelial cells. We found that mDia1 and Rac signaling are essential for the assembly of tangential AJs only in nontransformed epithelial cells. In contrast, Rac activity and mDia1 are not required for the assembly of radial AJs in transformed epithelial cells and fibroblasts. Earlier, Braga et al. [30] proposed that E-cadherin may be regulated by Rac differentially depending on cellular background (physiological context). Our data demonstrate the involvement of Rac signaling in the establishment of cadherin-mediated adhesion depending on the spatial organization of AJs. We propose that these differences are related to differential regulation of actin polymerization in the areas of tangential and radial AJs. Regulation by Rac1 and mDia1 of actin polymerization in tangential AJs is important for their establishment and stability.

We also showed that ROCK-stimulated myosin II activity is not required for the formation of tangential AJs in nontransformed epithelial cells. These findings confirmed the data of Sahai and Marshall [21] and Ivanov et al. [12], which established that myosin II motor activity is not essential for AJ assembly in epithelial MDCK and T84 cells. In contrast, myosin II-mediated contractility is a key regulator of the formation of radial AJs in transformed epithelial cells. As we and others discovered earlier, the formation of radial AJs in fibroblasts depends on myosin II-mediated contractility [26], [27]. Radial AJs in transformed epithelial cells are very similar to radial AJs in fibroblasts. Our study involving live-cell imaging of GFP-E-cadherin in transformed epithelial cells showed that AJs initially appear as dot-like clusters that grow into puncta or radial strands. We propose that the maturation of initial cadherin-based dot-like clusters into radial junctions depends on (i) association of an adhesion plaque with actin filaments, (ii) myosin II and α-actinin cross-linking of actin filaments into straight actin bundles, and (iii) centripetal myosin II-mediated tension of straight actin bundles, leading to growth of radial AJs. We also propose that, regulated by mechanical forces, actin polymerization like the recently described force-dependent actin polymerization in focal contacts [31] plays an important role in the formation of radial AJs. Local balance between myosin II-mediated contractile forces, adhesive interaction of cells with neighboring cells, and the generation of traction in the direction of lamellipodia extension contribute to growth, adhesion strengthening, and disruption of radial AJs.

**Figure 11:**
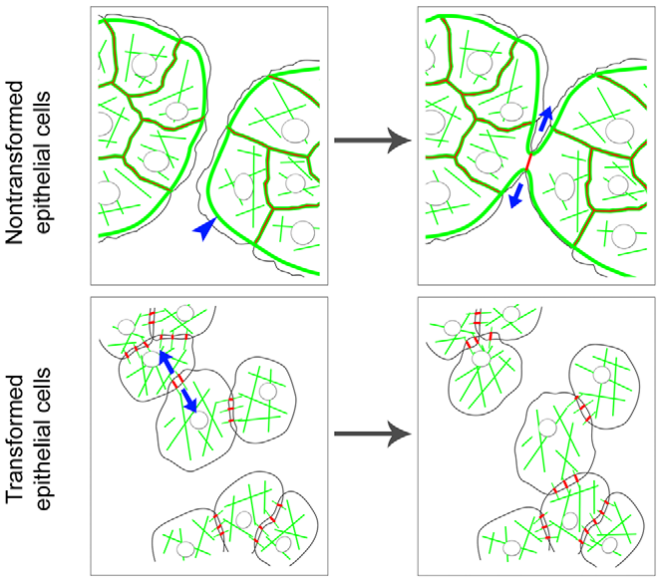
Hypothetical scheme for alterations of cell–cell interactions between epithelial cells at the early stages of morphologic transformation. The actin cytoskeleton (green) and AJs (red) in nontransformed and transformed epithelial cells. The characteristic feature of the actin cytoskeleton in nontransformed epithelial cells is marginal actin bundles at the free edges (blue arrowhead). In nontransformed cells, the formation of a tangential E-cadherin-based cell-cell contact (red line) leads to the disruption of marginal bundles at the site of the contact. The remaining segments of marginal bundles of adjacent cells form two arc-like bundles. The tangential tension of arcs (blue arrows) decreases protrusive activity both at the site of the cell-cell contact (contact paralysis) and at the free edges of contacting cells. In transformed cells, the crucial alteration of the actin cytoskeleton is the disappearance of marginal actin bundles. Upon the formation of the cell-cell contact, the deficiency of tangential tension as a result of the disappearance of marginal bundles prevents contact paralysis and leads to overlapping of lamellae of contacting cells. Radial AJs assemble in the area of overlapping. These AJs are associated with straight actin bundles. The enlargement of radial AJs depends on centripetal myosin II-mediated tension (blue arrows). AJs in transformed cells are very dynamic and unstable. Reorganization of the actin cytoskeleton and remodeling of AJs caused by neoplastic transformation results in a decrease in cell-cell adhesion and alters motile behavior of epithelial cells.

We hypothesize that the rearrangement of AJs in epithelial cells during neoplastic transformation into radial AJs is defined by reorganization of the actin cytoskeleton, leading to alterations of directions of tension in the area of cell-cell contacts. As we found earlier [32], the formation of tangential E-cadherin-mediated cell-cell contact in nontransformed epithelial cells leads to disruption of marginal bundles at the site of contact and to inhibition of retrograde actin flow. The remaining segments of marginal bundles of adjacent cells form two arc-like bundles (Figure 11) containing myosin II [33], which produce tangential tension in the zone of cell-cell contact in nontransformed epithelial cells. In transformed epithelial cells, disappearance of the marginal actin bundle is a key event that results in the rearrangement of AJs. In these cells, the deficiency of tangential tension and centripetal tension in the zone of cell-cell contact leads to the assembly of straight actin bundles and to the maturation of radial AJs.

In the present study using live-cell imaging, we also revealed significant alterations of cell-cell interactions caused by neoplastic transformation. The formation of stable contact between nontransformed epithelial cells results in dramatical inhibition of protrusions at the site of contact (contact paralysis) and in a decrease of protrusive activity at the free edges of contacting cells. In contrast, contact paralysis is not seen in transformed epithelial cells. Analysis of kymographs showed that lamellipodia at the sites of the cell-cell contacts continued to extend. Transformed cells also form lamellipodia at the free edges. We suggest that tangential tension of arc-like bundles in nontransformed epithelial cells may decrease the extension of protrusions both at the site of the cell-cell contact and at the free edges of contacting cells. Inhibition of protrusive activity stabilizes the contact between two cells. Similar suppression of protrusive activity after the application of artificial tangential tension with a micromanipulating needle was described by Kolega [34]. Tangential tension also can cause fast lateral expansion of contact and align newly assembled actin filaments in perijunctional bundles, strengthening the adhesion. The role of marginal actin bundles in the creation of tangential tension and the decrease of protrusive activity of contacting nontransformed epithelial cells was also supported by our experiments with Y-27632 and blebbistatin, which abolish marginal bundles. In the presence of these inhibitors, contact paralysis during the formation of cell-cell contact was not developed. We propose that the absence of marginal bundles in transformed epithelial cells leading to the deficiency of tangential tension at the border of contacting cells prevents inhibition of protrusive activity of contacting cells. The experiments performed suggest that the alterations in cell-cell interactions and the remodeling of AJs change motile behavior and migratory activity of transformed epithelial cells.

It should be noted that the cell-cell interactions between transformed epithelial cells described in this paper are very similar to those between cells of mesenchymal origin (fibroblasts). Earlier, we described the absence of contact paralysis and significant overlapping between contacting fibroblasts followed by persisting formation of lamellipodia at the free edges [26]. The formation of E-cadherin-based AJs in transformed epithelial cells and their regulation by Rho GTPases is similar to those in fibroblasts. Possibly, these alterations in transformed epithelial cells are manifestations of the initial stages of EMT. This question obviously needs further investigation.

Thus, our studies demonstrate that structural and dynamic coordination between cadherins, actin structures, and actin-regulating proteins can change during neoplastic transformation. In summary, we suggest a hypothetical scheme for the rearrangements of AJs and the actin cytoskeleton at the early stages of morphological transformation (Figure 11). These alterations are initiated by disruption of the marginal actin bundle. The deficiency of tangential tension as a result of the disappearance of the marginal bundle prevents contact paralysis and leads to overlapping of contacting cells. Dot-like AJs form in the zone of overlapping. Centripetal myosin II-mediated tension converts them into radial AJs, which are very dynamic and less stable than tangential AJs in nontransformed epithelial cells. Changes in the activities of Rho family small GTPases, which are characteristic of transformed cells, can be involved in these rearrangements. Remodeling of AJs and alterations of protrusive activity modulate cell-cell adhesion and motile behavior of cells. It is tempting to speculate that rearrangements of the actin cytoskeleton and of E-cadherin-based AJs observed in the present study may be essential for carcinoma cell dissemination.

## Materials and Methods

### Reagents

The ROCK inhibitor Y-27632 and the myosin II ATP-ase inhibitor (±)-blebbistatin were purchased from Calbiochem-Merck. The following primary monoclonal antibodies were used: anti-E-cadherin, clone 36; anti-N-cadherin, clone 32; anti-mDia1, clone 51 (Transduction Laboratories-BD); and anti-α-tubulin, clone DM 1A (Sigma-Aldrich). Secondary TRITC-conjugated antibodies were obtained from Chemicon; secondary Alexa488-conjugated antibodies; and Alexa488- and TRITC-conjugated phalloidin were obtained from Molecular Probes-Invitrogen. Secondary anti-mouse IgG_1_ and IgG_2a_ antibodies were purchased from Southern Biotech. Horseradish peroxidase-conjugated secondary antibodies for Western blotting were purchased from Upstate-Millipore. Other reagents were obtained from Sigma-Aldrich.

### Cell lines, constructs, and transfections

The IAR-2 line of nontransformed rat epithelial cells and the IAR-6-1 line of IAR epithelial cells transformed with dimethylnitrosamine were established by Montesano et al. [35]. The pPS/hygro-N-RasG12D construct was obtained from B.P. Kopnin (N.N. Blokhin Cancer Research Center, Russia). We obtained the IAR1162 and IAR1170 lines, expressing N-RasG12D, using retroviral infection from IAR-2 cells according to the procedure of retroviral transduction and selection with hygromycin [36]. We established individual clones of Ras-expressing IAR1162 cells (nine clones) and IAR1170 cells (five clones). Cells were maintained in Dulbecco's modified Eagle's medium (DMEM) supplemented with 10% fetal bovine serum, penicillin, and streptomycin at 37°C in a 5% CO_2_ humidified atmosphere. E-cadherin- and GFP-E-cadherin-expressing constructs were kindly provided by Dr. S.M. Troyanovsky (Northwestern University, Chicago, USA). We stably transfected Rat-1 fibroblasts with E-cadherin plasmid using the FuGene6 transfection reagent according to the manufacturer's protocol (Roche). To generate IAR-2 and IAR-6-1 lines that stably expressed GFP-E-cadherin, we transfected IAR-2 and IAR-6-1 cells with GFP-E-cadherin construct using the FuGene6 reagent. Stable transfectants were established after 2 weeks of selection in medium with G-418 (1 mg/ml).

### Analysis of formation of cell–cell contacts in a narrow wound

Cell-cell collisions and the establishment of cell-cell contacts were investigated in narrow wounds. We created narrow wounds by scraping cell monolayers on coverslips with an injection needle. Cultures were incubated for 3–4 h and observed or fixed at the time when the wound started to close. Y-27632 and blebbistatin were added to the growth medium from stock solutions at 20 min after wounding.

To evaluate the effects of Rho and Rac inhibition on the establishment of cadherin-mediated AJs in a narrow wound, C3 transferase and dominant negative N17Rac (kindly provided by Dr. A. Hall, Memorial Sloan-Kettering Cancer Center, USA) were used. Recombinant proteins were prepared as glutathione S-transferase fusion proteins in *Escherichia coli*, purified with glutathione Sepharose beads, thrombin cleaved, dialyzed against microinjection buffer, and concentrated as described previously [37]. C3 transferase and N17Rac were introduced into wound edge cells at the time of wounding, as a modification of the method of Brock et al. [38]. TRITC-labeled 10,000-MW lysine-fixable dextran (Molecular Probes) was used as a marker of loaded cells. Coverslips with cell monolayers were transferred into new dishes. Aliquots (10 µl) of protein solution (0.5 mg/ml) mixed with 1 µl of dextran were added on the monolayer surface. Monolayers were wounded with an injection needle, rinsed with warm DMEM, and incubated in DMEM before fixation with 3.7% paraformaldehyde (PFA).

### RNA interference

To deplete mDia1 in cells, we used the RNAi technique. The small interfering RNA oligonucleotides, corresponding to two mDia1 sequences from positions 795 to 813 and 184 to 209, were described previously [29] and chemically synthesized by Syntol (Moscow, Russia). Synthetic siRNA duplexes based on sense and antisense sequences were prepared as described [39]. GFP siRNA was used as negative control. We transfected mDia1 siRNA or control siRNA duplex oligonucleotides into cells using Lipofectamine 2000 according to the manufacturer's protocol (Invitrogen). The cultures were incubated for 36–48 h and analyzed with Western blotting or fixed after wounding of cell monolayers and establishment of new cell-cell contacts.

### Cell migration assay

We determined the migratory capacity of IAR cells *in vitro* using BD Bio-CoatTM migration chambers containing polycarbonate membrane inserts with 8-µm pores in a 24-well tissue culture plate (Becton Dickinson). Cells (2×10^4^ cells/200 µl of DMEM with 8% FCS) were added in the upper wells of chambers. The bottom well of each chamber was filled with DMEM with 8% FCS. After 20 h of incubation at 37°C with 5% CO_2_, cells from the upper surface of membranes were completely removed with a cotton swab; the migratory cells on the lower surface of membranes were fixed with 100% methanol and stained with DAPI. Membranes were mounted onto glass slides and examined microscopically at 20× magnification. We determined cellular migration by counting the numbers of stained cells on membranes in 15 randomly selected fields. The means of triplicate assays for each cell line in three independent experiments were used.

### Nude mouse tumorigenicity assay

Cells were resuspended in DMEM (1 million cells/300 µl DMEM) and were injected subcutaneously into the right rear flank of athymic nude (nu/nu) mouse (eight mice per group). Animals were monitored weekly for appearance and measurement of tumors. Tumor volumes (V) were determined from the three dimensions (height (a) by width (b) by depth (c) of tumors) (in millimeters) and calculated as V = 1/6πabc according to [40]. Animals were sacrificed 4 weeks postinjection, and the tumors, where present, were analyzed histologically and weighed.

### Fluorescence staining and microscopy

For E- or N-cadherin staining, cells were fixed with methanol-acetone (1∶1) at −20°C for 10 min. For double staining of E- or N-cadherin and of actin, cells were fixed with 1% PFA and permeabilized with 0.5% Triton X-100 for 5 min. To visualize mDia1, cells were fixed with 3.7% PFA and permeabilized. In some experiments, fixed cells were heated to 100°C and transferred into fresh PBS. Fixed specimens were incubated for 40 min with primary antibodies and then for 40 min with TRITC- or Alexa488-conjugated secondary antibodies. Alexa488- or TRITC-conjugated phalloidin was added to secondary antibodies. Fixed samples were examined with a Zeiss Axioplan microscope equipped with a 100× (NA 1.4) objective. Images were acquired with a cooled CCD ORCA-ER camera (Hamamatsu Photonics) using Wasabi 1.5 software.

We measured the intensity of E-cadherin and N-cadherin fluorescence at cell-cell contacts using ImageJ software (NIH, USA) as described [23]. The average of peak pixel intensity values in square segments (10×10 pixels) covering the entire cell-cell contact length was taken as a measure of fluorescence intensity at cell-cell contacts. For the quantification of cadherin accumulation at cell-cell contacts, we calculated a localization index by subtracting the peak value of cytoplasmic intensity from the peak value at the cell-cell contact and dividing this difference by the peak value of cytoplasmic intensity. This index was used to calculate relative fluorescence intensity. Control values were taken as 1.

### Live-cell imaging and image analysis

For live-cell imaging, cells were transferred into phenol red free α-MEM medium (Gibco, Invitrogen) supplemented with 10% FCS and were observed with a Nikon Eclipse-Ti microscope equipped with a Plan-Neofluar 100× (NA 1.3) DIC objective at 35°C. Images were acquired with an ORCA-ER Hamamatsu camera controlled with NIS-Elements AR2.3 software (Nikon). In some cases, we used Leica TCS SP5 confocal laser scanning microscope. We measured overlapping areas as described [32], using SimplePCI 5.3.1 software (Hamamatsu). To analyze lamellipodial dynamics in the narrow wound, time-lapse sequences were acquired with 3-s intervals for 25–30 min. Using ImageJ software (NIH, USA), we generated kymographs along straight lines drawn in the direction of protrusion. Rates of lamellipodial protrusion and retraction were determined on the basis of slopes produced by advancing leading edges. The mean rates during 5 min periods of observation were used.

Using ImageJ software, we measured the distances traveled by individual AJs and calculated the mean rates of AJ movement. The distance map method was used to study the dynamics of AJs. We analyzed selected frames from confocal images of GFP-E-cadherin in living cells in sparse cultures using ImageJ software as described [25]. Binary images produced from grey images were thinned until they formed binary skeletons, which represent central positions of AJs. Distance maps (D*i*) were created; these are images of the distance to the nearest point from the skeleton. Next, the binary skeletons at times *i*+1 were overlaid on the distance maps (D*i*). In the absence of movement, the pixel value on the distance map below the skeleton is zero. If movement occurred between the two images, the pixel value below the skeleton indicates the distance traveled between times *i* and *i*+1. The average value of the pixel-to-pixel multiplication of S*i*+1 and D*i* quantifies the average displacement of AJs between two successive images.

### Western blotting

SDS-PAGE and Western blotting were performed in minigel PAGE devices (Mini-Protean® 3 Cell, Bio-Rad). For Western blotting of whole cell lysates, cells were lysed with RIPA buffer (50 mM Tris-HCl, pH 6.8; 2% SDS; 16% glycerol; 1% mercaptoethanol; 0.004% bromphenol blue) including protease inhibitor cocktail (Sigma). Samples (10 or 20 µl) were heated for 5 min at 95°C and used immediately for 10% SDS-PAGE in equal protein concentrations according to the Bio-Rad protocol. Resolved proteins were transferred to PVDF membranes, which were then probed with appropriate primary and peroxidase-conjugated secondary antibodies. We detected blotted protein bands using an ECL Plus kit (Amersham), and we captured chemiluminescence images using an image acquisition Chemi-Smart 2000 system (Vilber Lourmat) and Chemi Capt software.

## Supporting Information

Figure S1 Expression of E-cadherin and N-cadherin in nontransformed IAR-2 cells and transformed IAR-6-1, and IAR1170 cells. Total cell lysates were resolved by means of SDS-PAGE and immunoblotted for E-cadherin (A), N-cadherin (B), and α-tubulin (as loading control). Nontransformed IAR-2 cells express epithelial E-cadherin. Transformed IAR-6-1 cells express E-cadherin and do not express N-cadherin. Transformed IAR1170 cells retain E-cadherin and also express N-cadherin.
(1.05 MB TIF)

Figure S2 Tumorigenic effects of the injection of transformed IAR-1170 cells (clone H5). Cells were injected subcutaneously into athymic nude mice. (A) Tumor volume was measured as described in Materials and Methods. (B) Tumor weight was determined at 4 weeks postinjection, when animals were sacrificed. Each value is the mean±SEM of eight determinations. Tumors were not formed after injection of nontransformed IAR-2 cells.
(2.18 MB TIF)

Figure S3 Kymographs generated at the free edges of contacting cells (left) were used to quantifiy the rates of lamellipodial protrusion and retraction (right). In nontransformed IAR-2 cells, the establishment of cell-cell contacts resulted in the decrease of the rates of lamellipodial protrusion at the free edges (from 4.0 µm/min to 2.6 µm/min) (***, p<0.05, t-test). In transformed IAR-6-1 cells, the rates of lamellipodial protrusion and retraction at the free edges of contacting cells did not change within the time of observation. White line on kymograph indicates the time of the establishment of the contact. Data are presented as means±SEM.
(1.40 MB TIF)

Figure S4 Schematic representation of the distance map method for measurement of the rate of AJ movement. (A and B) Fragments of confocal images of AJs at times i and i +3 min (image size, 34 µm). Images were acquired at 3-min intervals. (C and D) Skeleton images corresponding to images A and B. (E) Map of the distances from the skeleton at time i. (F) Result of the multiplication of images D and E. The intensity of each pixel in image F corresponds to the distance in pixels traveled by AJs during 3 min.
(0.43 MB PDF)

Figure S5 In the presence of Y-27632, the establishment of the contact between IAR-2 cells is not accompanied by contact paralysis. Left: kymograph generated at the site of the cell-cell contact. White line indicates the time of the establishment of the contact. Right: quantification of the rates of lamellipodial protrusion and retraction at the sites of cell-cell contacts.
(0.46 MB PDF)

Video S1 Stable cell-cell contacts in an IAR-2 island. Cell-cell contacts are stable during the entire period of observation (Re: Figure 2A). Images were acquired at 5-min intervals for 195 min.
(1.17 MB MOV)

Video S2 Cell-cell interactions in a sparse culture of transformed IAR-6-1 cells. Cells extend lamellipodia at different sites along free edges and establish contacts with different neighboring cells. Cell-cell contacts are unstable and often break. Cells move in different directions (Re: Figure 2B). Images were acquired at 5-min intervals for 360 min.
(2.63 MB MOV)

Video S3 Cell-cell interactions in a sparse culture of transformed IAR1170 cells (clone H5). The cells establish dynamic contacts with neighboring cells. Cell-cell contacts are unstable and may break (Re: Figure 2C). Images were acquired at 5-min intervals for 425 min.
(3.42 MB MOV)

Video S4 Dynamics of cell-cell interactions between nontransformed IAR-2 cells in a narrow wound. By 15-20 min, protrusive activity at the contact site is inhibited (contact paralysis). At the same time, the extension of lamellipodia at the free edges of contacting cells decreases (Re: Figure 4A). Images were acquired at 3-s intervals for 20 min.
(10.15 MB MOV)

Video S5 Dynamics of cell-cell interactions between transformed IAR-6-1 cells in a narrow wound. Upon formation of the contact, protrusions extend both at the site of the contact (absence of contact paralysis) and at the free edges (Re: Figure 4C). Images were acquired at 15-s intervals for 28 min.
(4.28 MB MOV)

Video S6 GFP-E-cadherin dynamics at the sites of cell-cell contacts in a sparse IAR-2 culture. Nontransformed IAR-2 cells were stably transfected with GFP-E-cadherin. GFP-E-cadherin accumulates in the zones of cell-cell interactions, forming stable tangential AJs (Re: Figure 5A). Images were acquired at 10-min intervals for 170 min.
(0.27 MB MOV)

Video S7 Remodeling of AJs in transformed IAR-6-1 cells. Transformed IAR-6-1 cells were stably transfected with GFP-E-cadherin. Upon establishment of cell-cell contact, E-cadherin aggregates in dot-like AJs at the site of the contact. AJs can grow, rearrange, and relocate in the area of the contacts. The disruption of AJs results in detachment of one cell from another (Re: Figure 5B). Images were acquired at 10-min intervals for 320 min.
(0.64 MB MOV)

Video S8 Accumulation of GFP-E-cadherin at the contact between two nontransformed IAR-2 cells in a narrow wound. During the formation of the cell-cell contact, GFP-E-cadherin accumulates in a tangential line at the boundary between two cells (Re: Figure 6A). Images were acquired at 30-s intervals for 13 min.
(0.81 MB MOV)

Video S9 GFP-E-cadherin dynamics at the site of the cell-cell contact of transformed IAR-6-1 cells in a narrow wound. GFP-E-cadherin initially accumulates in small, dot-like clusters. A majority of E-cadherin dots transform into radial strands. Within the time of observation, the size of radial AJs can grow (Re: Figure 6B). Images were acquired at 30-s intervals for 47 min.
(0.98 MB MOV)
