# Supplementary figures and images for: Rearrangements of the Actin Cytoskeleton and E-Cadherin–Based Adherens Junctions Caused by Neoplasic Transformation Change Cell–Cell Interactions
Source: PLoS One. 2009 Nov 30;4(11):e8027. doi: 10.1371/journal.pone.0008027 (PMC2779654; doi:10.1371/journal.pone.0008027)

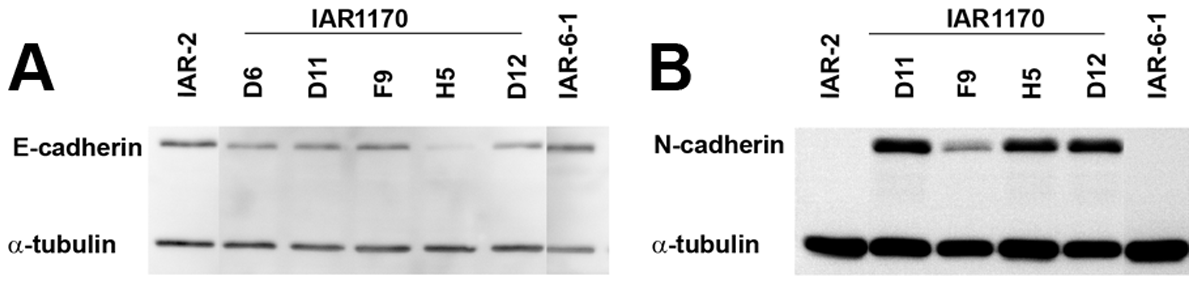

Supplement: Figure S1 — Expression of E-cadherin and N-cadherin in nontransformed IAR-2 cells and transformed IAR-6-1, and IAR1170 cells. Total cell lysates were resolved by means of SDS-PAGE and immunoblotted for E-cadherin (A), N-cadherin (B), and α-tubulin (as loading control). Nontransformed IAR-2 cells express epithelial E-cadherin. Transformed IAR-6-1 cells express E-cadherin and do not express N-cadherin. Transformed IAR1170 cells retain E-cadherin and also express N-cadherin. (1.05 MB TIF) [file pone.0008027.s001.tif]

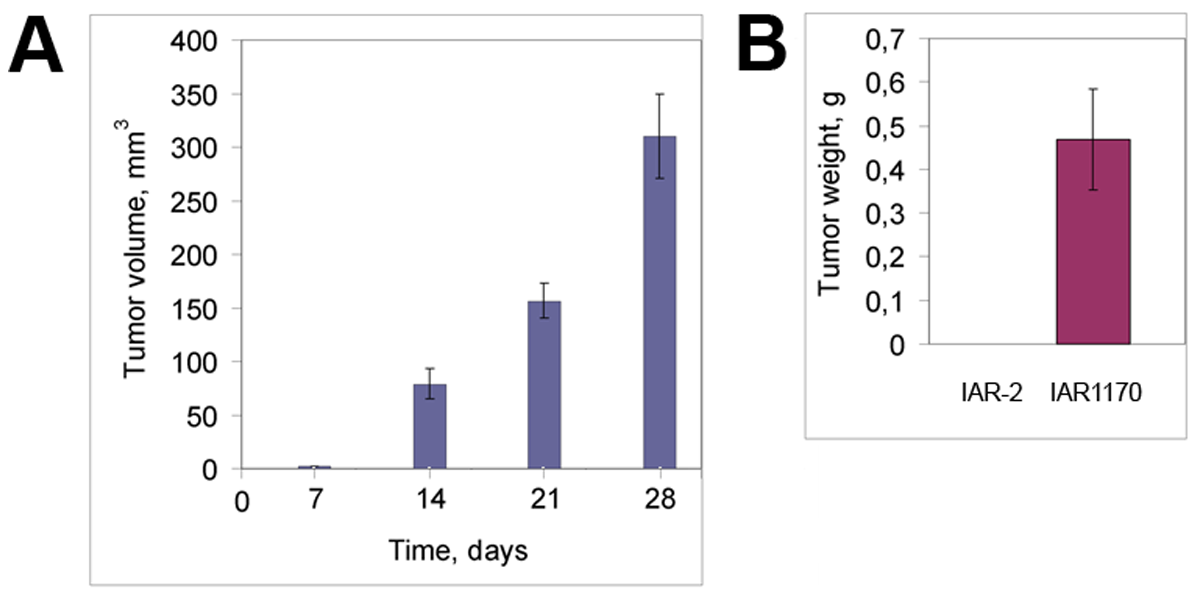

Supplement: Figure S2 — Tumorigenic effects of the injection of transformed IAR-1170 cells (clone H5). Cells were injected subcutaneously into athymic nude mice. (A) Tumor volume was measured as described in Materials and Methods. (B) Tumor weight was determined at 4 weeks postinjection, when animals were sacrificed. Each value is the mean±SEM of eight determinations. Tumors were not formed after injection of nontransformed IAR-2 cells. (2.18 MB TIF) [file pone.0008027.s002.tif]

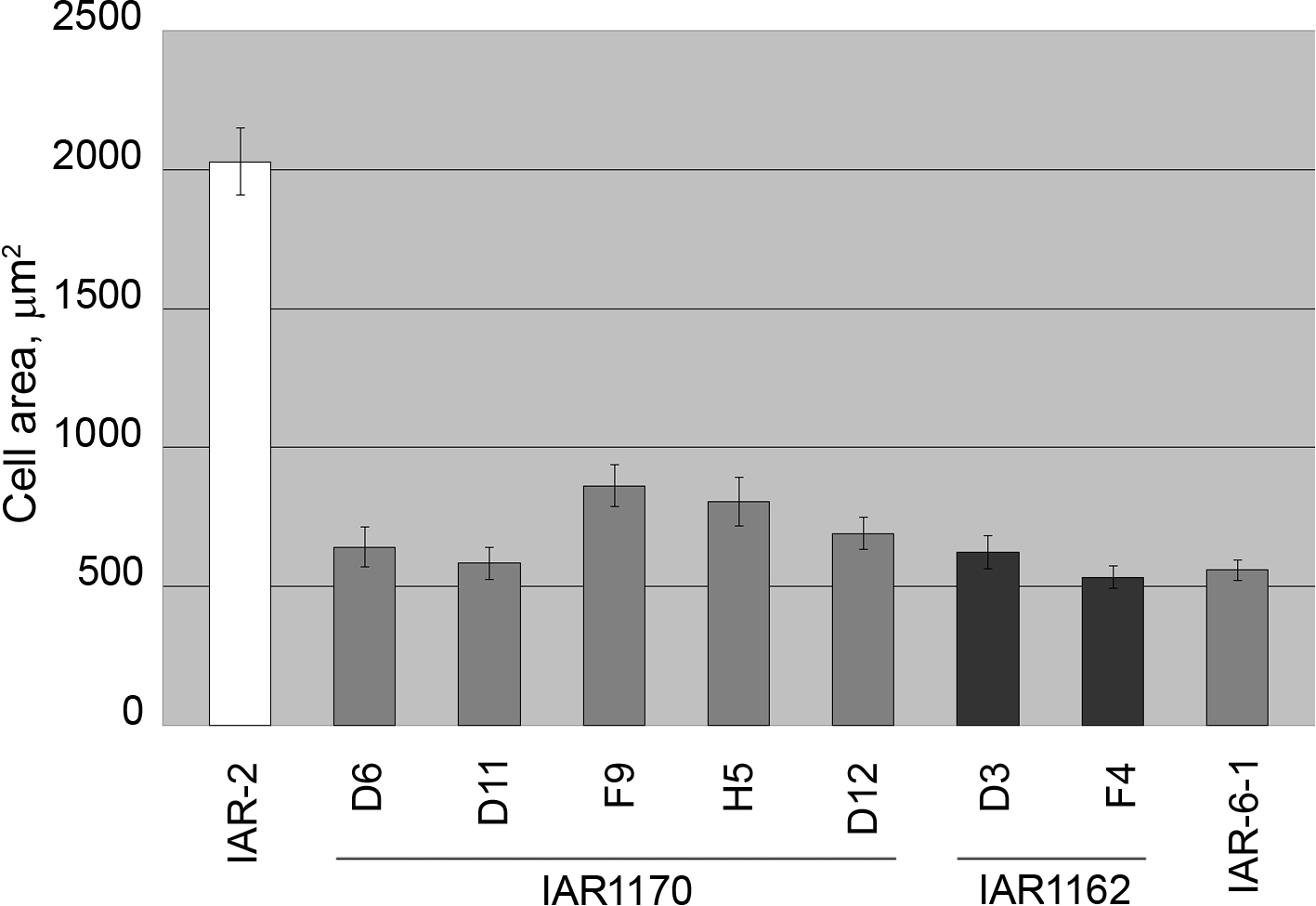

Supplement: Figure S3 — Kymographs generated at the free edges of contacting cells (left) were used to quantifiy the rates of lamellipodial protrusion and retraction (right). In nontransformed IAR-2 cells, the establishment of cell-cell contacts resulted in the decrease of the rates of lamellipodial protrusion at the free edges (from 4.0 µm/min to 2.6 µm/min) (***, p<0.05, t-test). In transformed IAR-6-1 cells, the rates of lamellipodial protrusion and retraction at the free edges of contacting cells did not change within the time of observation. White line on kymograph indicates the time of the establishment of the contact. Data are presented as means±SEM. (1.40 MB TIF) [file pone.0008027.s003.tif]

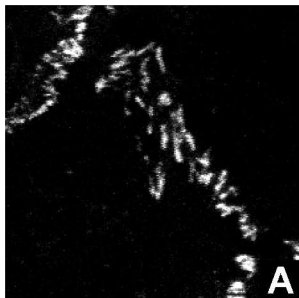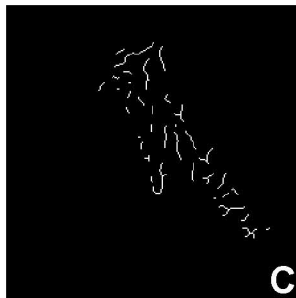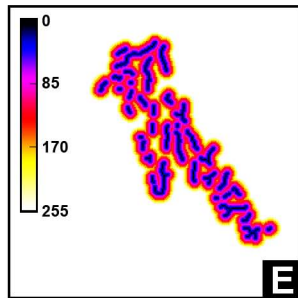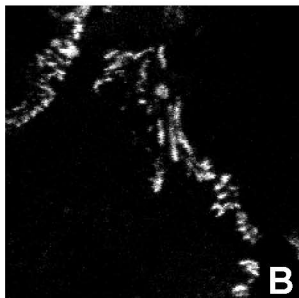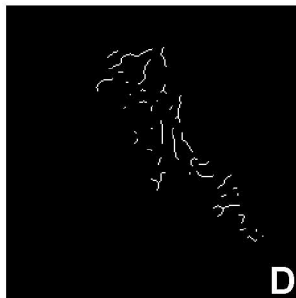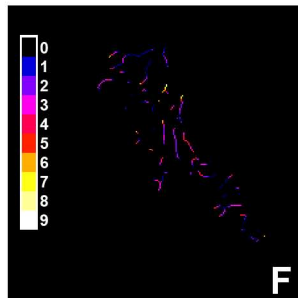

Supplement: Figure S4 — Schematic representation of the distance map method for measurement of the rate of AJ movement. (A and B) Fragments of confocal images of AJs at times i and i +3 min (image size, 34 µm). Images were acquired at 3-min intervals. (C and D) Skeleton images corresponding to images A and B. (E) Map of the distances from the skeleton at time i. (F) Result of the multiplication of images D and E. The intensity of each pixel in image F corresponds to the distance in pixels traveled by AJs during 3 min. (0.43 MB PDF) [file pone.0008027.s004.pdf]

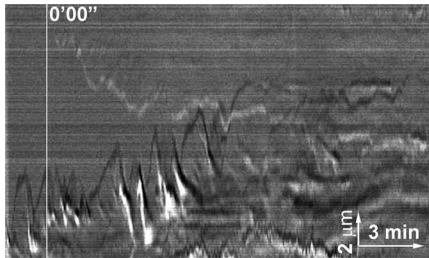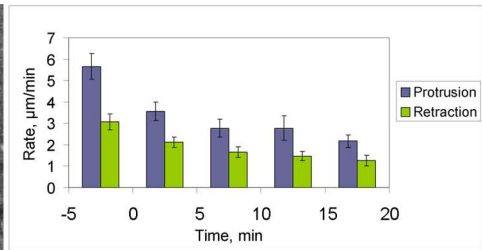

Supplement: Figure S5 — In the presence of Y-27632, the establishment of the contact between IAR-2 cells is not accompanied by contact paralysis. Left: kymograph generated at the site of the cell-cell contact. White line indicates the time of the establishment of the contact. Right: quantification of the rates of lamellipodial protrusion and retraction at the sites of cell-cell contacts. (0.46 MB PDF) [file pone.0008027.s005.pdf]
